# Congenital Zika syndrome and neuroimaging findings

**DOI:** 10.1590/0100-3984.2018.51.2e2

**Published:** 2018

**Authors:** Bruno Niemeyer de Freitas Ribeiro

**Affiliations:** 1 Masters Student, Universidade Federal do Rio de Janeiro (UFRJ), MD, Neuroradiologist at the Instituto Estadual do Cérebro Paulo Niemeyer, Rio de Janeiro, RJ, Brazil. E-mail: bruno.niemeyer@hotmail.com

The Zika virus (ZIKV) is an arbovirus of the Flaviviridae family, composed of RNA; it was first identified in 1947 in the Zika forest in Uganda in rhesus monkeys, which were serving as sentinels for yellow fever surveillance, and the first human case was reported in 1952^(1-5)^. Typically, ZIKV occurs in tropical and subtropical areas of the world; there are two recognized strains-African and Asian-and three recognized genotypes-West African, East African, and Asian-the Asian genotype being responsible for the outbreak in Brazil^(1-5)^.

In most cases, transmission of ZIKV occurs through the bite of mosquitoes of the genus *Aedes*, primarily *Aedes aegypti*, mosquitoes of other genera being potential vectors for the spread of the infection^(1-5)^. Other forms of transmission have been described, including blood transfusion, sexual contact, and contact with infected urine, as well as transplacental and perinatal transmission^(1-5)^.

Most ZIKV infections are asymptomatic (80%); when symptomatic, they are commonly characterized by a self-limited condition including maculopapular rash, low-grade fever, headache, arthralgia, and nonpurulent conjunctivitis, the symptoms typically regressing within two to seven days^(1-5)^. Although rare, central nervous system (CNS) involvement in ZIKV infection has been reported, including cases of meningoencephalitis, Guillain-Barré syndrome, and acute disseminated encephalomyelitis^(1-7)^. The diagnosis of ZIKV infection can be confirmed by amplification of the viral genome with reverse transcriptase-polymerase chain reaction in biological samples (blood, saliva, urine, cerebrospinal fluid, or amniotic fluid) or by serological tests for the detection of immunoglobulin M antibodies against ZIKV^(1-6)^.

The treatment of ZIKV infection is directed at the symptoms, given that there are as yet no vaccines or antiviral therapies. The main focus has been on prevention, including vector eradication and warnings against travel to endemic areas.

Although previous ZIKV outbreaks occurred in Micronesia in 2007 and in French Polynesia in the 2013-2014 period, the association between congenital ZIKV infection and CNS malformations was recognized only in October 2015, when there was a dramatic increase in the number of cases of microcephaly in northeastern Brazil^(1,3-5,8,9)^. In February 2016, the World Health Organization declared the relationship between ZIKV infection and congenital malformations an international health emergency^(9)^.

Within Latin America, Brazil was the country most affected by the ZIKV epidemic, approximately 1,500,000 cases being reported between 2015 and 2016, during which time the incidence of microcephaly increased by approximately 20 times in comparison with previous years^(1,8,9)^. The number of reported cases of ZIKV infection in Brazil has subsequently decreased, from approximately 205,000 cases in 2016 to approximately 13,000 cases in 2017, population immunity being considered the main reason for this decline^(9)^. However, the continuous transmission, in Brazil and in the Americas at large, of the four dengue serotypes in recent decades suggests that ZIKV will continue to circulate, making it necessary for physicians to suspect ZIKV infection when there is a clinical profile consistent with the diagnosis^(9)^.

The manifestations of intrauterine ZIKV infection are more severe when they occur in the first or second trimesters of gestation, ranging from various congenital abnormalities-redundant skin on the scalp and neck (cutis gyrata), low birth weight, polyhydramnios, anasarca, arthrogryposis, and hearing loss, as well as ocular and CNS malformations-to fetal death^(1,2,10-18)^.

The article authored by Peixoto Filho et al.^(19)^, published in this issue of Radiologia Brasileira, clearly illustrates the main aspects of computed tomography and magnetic resonance imaging of the skulls of children with CNS malformations likely related to ZIKV infection. In that article, nine children were examined and the main imaging aspects were as follows: microcephaly; occipital protuberance with excessive scalp skin; reduced volume of the cerebral parenchyma, with gyral simplification; ventriculomegaly; parenchymal calcifications, predominantly located at the cortico-subcortical junction, agenesis/dysgenesis of the corpus callosum, and changes in the posterior fossa, such as cerebellar and pontine hypoplasia. Those aspects are consistent and corroborate findings reported in the literature^(1,10-18)^.

In conclusion, as presented by Peixoto Filho et al.^(19)^, the neuroimaging aspects of congenital ZIKV syndrome are becoming increasingly more consolidated. It is of fundamental importance for radiologists to have knowledge of those aspects because, although they are not pathognomonic, they can raise the diagnostic suspicion of the syndrome.
